# An Improved Melon Reference Genome With Single-Molecule Sequencing Uncovers a Recent Burst of Transposable Elements With Potential Impact on Genes

**DOI:** 10.3389/fpls.2019.01815

**Published:** 2020-01-31

**Authors:** Raúl Castanera, Valentino Ruggieri, Marta Pujol, Jordi Garcia-Mas, Josep M. Casacuberta

**Affiliations:** ^1^ Centre for Research in Agricultural Genomics CSIC-IRTA-UAB-UB, Campus UAB, Edifici CRAG, Barcelona, Spain; ^2^ Institut de Recerca i Tecnologia Agroalimentàries (IRTA), Genomics and Biotecnology Program, Barcelona, Spain

**Keywords:** long-reads, assembly, reference genome, transposable elements, melon

## Abstract

The published melon (*Cucumis melo* L.) reference genome assembly (v3.6.1) has still 41.6 Mb (Megabases) of sequences unassigned to pseudo-chromosomes and about 57 Mb of gaps. Although different approaches have been undertaken to improve the melon genome assembly in recent years, the high percentage of repeats (~40%) and limitations due to read length have made it difficult to resolve gaps and scaffold's misassignments to pseudomolecules, especially in the heterochromatic regions. Taking advantage of the PacBio single- molecule real-time (SMRT) sequencing technology, an improvement of the melon genome was achieved. About 90% of the gaps were filled and the unassigned sequences were drastically reduced. A lift-over of the latest annotation v4.0 allowed to re-collocate protein-coding genes belonging to the unassigned sequences to the pseudomolecules. A direct proof of the improvement reached in the new melon assembly was highlighted looking at the improved annotation of the transposable element fraction. By screening the new assembly, we discovered many young (inserted less than 2Mya), polymorphic LTR-retrotransposons that were not captured in the previous reference genome. These elements sit mostly in the pericentromeric regions, but some of them are inserted in the upstream region of genes suggesting that they can have regulatory potential. This improved reference genome will provide an invaluable tool for identifying new gene or transposon variants associated with important phenotypes.

## Introduction

Melon (*Cucumis melo* L.) is one of the most important plant crops, with a worldwide production reaching near 32 million tons in 2017 (http://www.fao.org). A high-quality reference genome assembly of melon was released in 2012 (Garcia-Mas et al., 2012). This assembly was generated using 454 reads and Sanger sequencing of BAC ends and contained up to 375 Mb of sequence assembled into 1,594 scaffolds, with an N50 of 4.68 Mb. Since then, additional improvements have been performed. In particular, a high-resolution genetic map was used to anchor up to 98.2% of the scaffold assembly to the 2X = 24 melon chromosomes (Argyris et al., 2015), followed by an optical mapping used to improve the orientation of the scaffolds of the previous assembly and accurately define the gap content (Ruggieri et al., 2018). Besides the efforts done to improve the original assembly, the lastest published melon reference genome (v3.6.1) (Ruggieri et al, 2018), still contains up to 19.1% of its sequence in gaps and 41.6 Mb of unassigned sequences (22,123 unassigned contigs out of the 42,067 contigs, grouped as Chr0).

Previous analyses on the melon genome architecture have described that this species contains expanded pericentromeres arising from massive amplification of transposable elements (TEs) in the past 10 million years (Mya) (Morata et al., 2018). TEs tend to accumulate in centromeric and pericentromeric regions due to the preferential insertion of some elements, including some retrotransposon families (Neumann et al., 2011), but also as a consequence of the counter-selection of insertions in genic regions that are more likely to be deleterious (Contreras et al., 2015). Due to the enriched proportion of long, repeated sequences such as Long Terminal Repeat (LTR) retrotransposons and other TEs, plant centromeres, and pericentromeres are difficult to assemble and often contain multiple gaps. Such difficulty arises from the limitation of the short-read sequencing to distinguish between near-identical repeated regions. However, this limitation will also make it difficult to correctly assemble TEs sitting in gene-rich regions. As a consequence, short-read-based assemblies may contain an underestimated transposon content, with elements missing in the pericentromeric regions, but also in the proximity of genes, and potentially impacting on gene regulation or coding capacity. Third generation sequencing offers a great opportunity to improve short-read-based assemblies such as the melon reference genome due to the longer read length, the low systematic bias, high consensus read accuracy, and improved assembling algorithms. In the recent years, many studies have taken advantage of these technologies for improving draft genomes or generating chromosome-level assemblies (Jiao et al., 2017b; Zhang et al., 2019). We describe here a new reference assembly for the cultivated melon (v4.0). This new version benefited from ~50-fold PacBio long reads coupled with a 20-fold Illumina short-reads data, which allowed to improve the characterization and accuracy of several regions of the genome, particularly repetitive regions and centromeric areas.

## Methods

### DNA Extraction and Sequencing

Genomic DNA was extracted from the double-haploid line DHL92, the same line sequenced to obtain the previous version of the melon genome, v3.6.1 (Ruggieri et al., 2018), as described by (Doyle, 1991) with minor modifications. Three grams of young leaves were harvested and frozen in liquid nitrogen for tissue homogenization. After isopropanol precipitation, instead of centrifugation, the DNA was recovered by fishing with a little glass hook to avoid fragmentation. We added a purification step using phenol:chloroform:isoamyl alcohol (25:24:1), and resuspended in Milli-Q^®^ water. DNA integrity was evaluated by gel electrophoresis and quantified by Qubit 2.0. DNA was purified with AMPure^®^ PB beads, and length was evaluated with the Fragment Analyzer Femto Pulse (Advanced Analytical Technologies, Inc.). DNA sequencing was performed using Pacific Biosciences (PacBio) RSII technology at the Platform GENTYANE, INRA/UCA (Clermont-Ferrand, France). A total of ∼2,5 million PacBio long reads were generated, which corresponds to ∼50x coverage of the estimated melon genome.

### Genome Assembly

The reads from the PacBio system were assembled using the hierarchical genome-assembly process 4 (HGAP4) pipeline (Pacific Biosciences, SMRT Link Suite 6.0). The principle and workflow of HGAP pipeline consists of different concatenated steps, including (i) the selection of the longest reads as a seeding sequence data set, (ii) the use of each seeding sequence as a reference to recruit shorter reads and preassemble reads through a consensus procedure, (iii) the assembly of the preassembled reads, (iv) the refinement/polishing using all initial read data to generate the final consensus (Chin et al., 2013). In the pre-assembly step, the raw reads were filtered using default settings with read quality (rq) of ≥ 0.65. Then, the assembly step was performed using FALCON in the HGAP4 tool with seed coverage set to 50, “aggressive” option turned on, and minimum accuracy set to 65. The ARROW algorithm was used to polish the genome assembly with default parameters.

### Reference-Guided Contig Ordering, Orientation, and Genome Quality Assessment

The contigs produced by the assembly were ordered and oriented based on the latest melon assembly (v3.6.1) with the RaGOO tool, which uses a reference-guided process (Alonge et al., 2019). In order to improve the mappability of PacBio contigs, a polishing step was previously performed on the v3.6.1 assembly using raw PacBio reads. With this aim, the ARROW pipeline in the SMRT Link suite (resequencing pipeline) was used with default parameters, just superimposing the minimum number of reads to call variant ≥ 15.

RaGOO is an open-source tool, implemented as a python command-line utility, which internally invokes Minimap2 (Li, 2018). Default parameters were used with k-mer size and window size both set to 19 bp. Any alignment shorter than 1 kbp in length was removed. As reported by the author, to cluster contigs, the tool assigns each contig to the reference chromosome which it covers the most. Subsequently, for each pseudomolecule group, the contigs in that group are ordered and oriented relative to each other by examining the longest (primary) alignment. Ordering is then achieved by sorting these primary alignments. To produce pseudomolecules, the contigs are concatenated, with padding of 1,000 “N” characters placed between contigs. Finally, the new consensus sequences were polished with 20× Illumina paired-end reads (2 × 150 bp). Reads were aligned to the assembly using BWA-MEM (Li and Durbin, 2009). Sequence error correction was performed with the Pilon pipeline (Walker et al., 2014). The completeness of the final assembly was evaluated with BUSCO (version 3) (Simão et al., 2015) using the conserved plant genes as database (Eudicotyledons odb10*). Comparative analysis and synteny between v3.6.1 and v4.0 assemblies were performed using MAUVE (Darling et al., 2004) and SyMAP v4.2 (Soderlund et al., 2011).

### Genome Annotation

The genome annotation was performed by transferring through a liftover process the latest published gene models (Ruggieri et al., 2018) to the new PacBio-based genome assembly using Maker v2 program (Campbell et al., 2014). The parameters used in the configuration file were the following: est_forward = 1, est2genome = 1, split_hit = 20000, min_intron = 20, single_exon = 1, single_length = 149, correct_est_fusion = 1. In case of a gene mapping on different positions of the genome, only the match with the highest Maker score was retained. Gene ontology (GO) enrichment analysis was carried out using GOATOOLS (Klopfenstein et al., 2018)

### Annotation of Transposable Elements

Transposable elements were detected in the new genome assembly using TEdenovo pipeline from the REPET package (Flutre et al., 2011), excluding the structural search. Consensus sequences representing each TE family were classified into TE orders using PASTEC (Hoede et al., 2014) and annotation of TE copies was carried out by TEannot using two iterations. After the first TEannot run, only consensus sequences that had a full-length match in the genome were retained. A second iteration of TEannot using these consensuses was used to obtain the final annotation. We used blastx (Repbase peptide database (Bao et al., 2015), cut off e-value = e-5) to identify TIR-TE copies that retained coding potential.

### Specific Annotation of LTR-Retrotransposons

LTR-retrotransposon candidates were detected by a structural approach using LTRharvest (Ellinghaus et al., 2008). Every element was translated to the six possible frames and scanned for LTR-retrotransposon-specific domains using hmmscan (Eddy, 2011). Elements without coding potential were filtered out, and the remaining elements were classified into Copia and Gypsy superfamilies based on the order of the internal coding domains, as defined by (Xiong and Eickbush, 1990). Elements lacking one or more domains were tagged as “unclassified”.

### Insertion Age of LTR-Retrotransposons

The LTR regions of every coding element were extracted and aligned with MUSCLE (Edgar, 2004). Kimura 2P distance of every aligned LTR pair was calculated and used to estimate insertion age as previously reported (SanMiguel et al., 1998), using the *Arabidopsis* mutation rate of 7x10^-9^ nucleotides per site per year (Ossowski et al., 2010).

### Identification of Polymorphic LTR-Retrotransposons

Resequencing short-read data from six melon varieties (CV, IRAK, PI 161375, Trigonous, Calcuta, and Vedrantais) were previously available (Garcia-Mas et al., 2012; Sanseverino et al., 2015). Trimming and adapter removal was performed with AdapterRemoval (Lindgreen, 2012). Clean reads were mapped to the v4.0 assembly using BWA-MEM (Li and Durbin, 2009). PINDEL (Ye et al., 2009) was run on mapping files to identify deletions in re-sequenced varieties, using the following parameters: Minimun mapping quality = 35, minimum number of supporting reads for calling a deletion = 5. A polymorphic insertion was scored when the deletion and reference element displayed a reciprocal intersect of 90% of the length.

## Results

### Genome Assembly Workflow

The approach followed relies on a combination of different pipelines and resources as highlighted in **Supplementary Figure S1**. The first step took as input four PacBio runs, which yielded about 21 Gigabases of sequence (corresponding approximately to a 50x melon genome coverage) with an average read length of 8 kbp and an N50 of about 15 kbp (**Supplementary Table S1**). At the pre-assembly stage, 1,499,406 seed reads were selected with an average length of 12 Kbp. The seed reads were used to produce about 1,469,624 pre-assembled reads (**Supplementary Table S1**). The final consensus assembly yielded 1,178 contigs with a N50 of 714 kbp and a total genome size of 357.64 Mbp. The final mean coverage obtained and the realigned subread concordance are illustrated in **Supplementary Figure S2**.

### Pseudomolecule Construction

Following a reference-guided process, the contigs produced by the assembly were ordered and oriented based on the latest melon assembly (v3.6.1) (**Supplementary Figure S1**). In order to improve the mappability of the PacBio contigs on the genome, a polishing/correction step using the complete set of PacBio reads was undertaken. A total of 648,906 variants (271,290 deletions, 293,163 insertions and 84,453 substitutions) on the published v3.6.1 genome were corrected, leading to 1% improvement in mapping of the PacBio contigs. During pseudomolecule construction, we could assign 96% of the contigs to the 12 melon chromosomes, leaving only 44 unassigned short contigs (average length of 7.2 kbp).

### Further Polishing of Pseudomolecules

**Figure 1:**
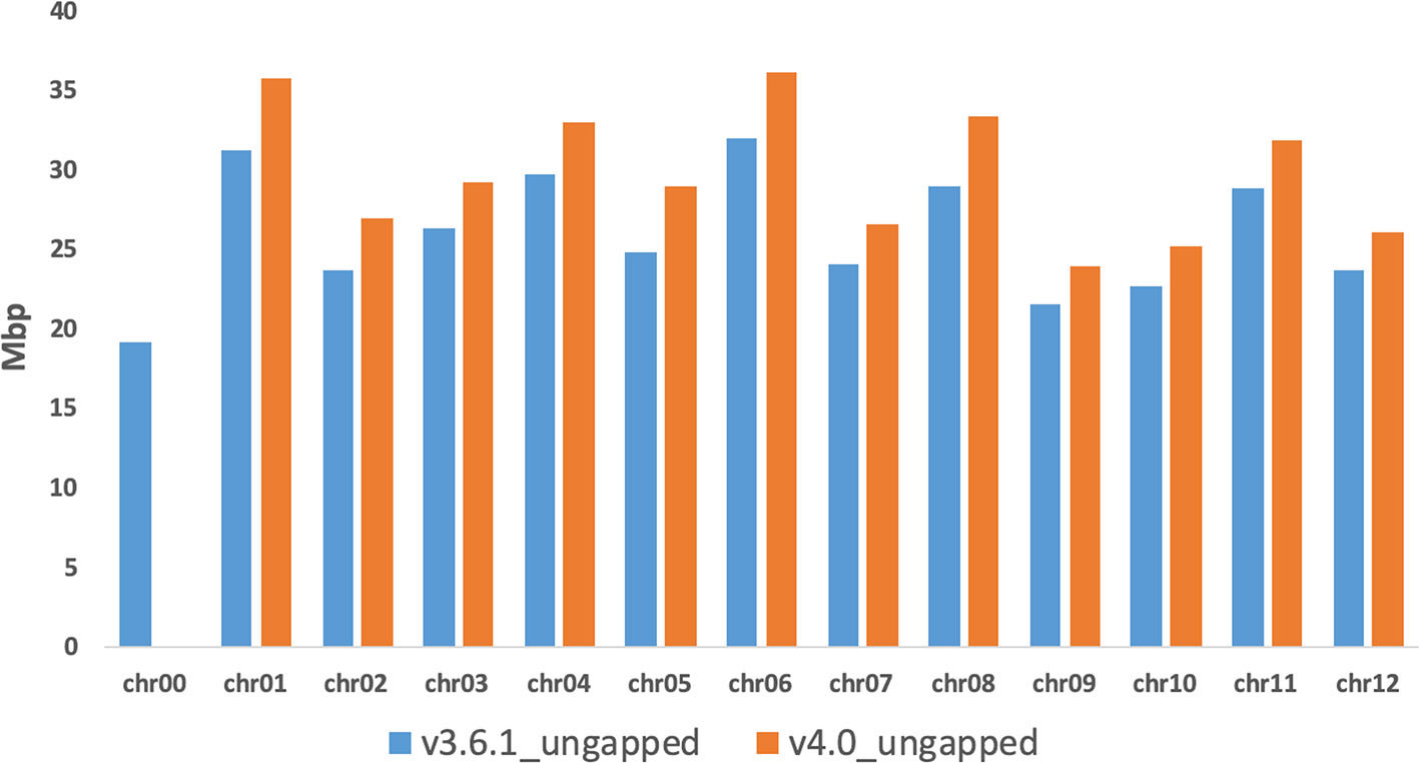
Comparison of the chromosomes length (ungapped) between the v3.6.1 and the v4.0 genome assemblies.

The last step of the workflow was aimed to correct/polish the PacBio assembly using 20-fold Illumina reads from a previous study (Sanseverino et al., 2015). A total of 169,279 variants (117,320 insertions, 29,353 deletions, 22,606 substitutions), representing less than 0.1% of the total genome size in length, were detected and included in the final genome v4.0. After error correction using short sequence reads, the total size of melon pseudomolecules is 358 Mb. The new reference assembly contains 1,169 artificial gaps (strings of 1,000 Ns) and has a much higher contiguity than the previously published short-read genome assembly DHL92 v3.6.1 (contig N50 improved from 26.1 kb to 714 kb; contig number improved from 42,067 to 1,178). **Figure 1** shows an improvement of the v4.0 genome assembly in terms of length increase of each chromosome and reduction of unassigned contigs in Chr0 reduction when compared with v3.6.1 assembly. These results highlight the increase of the pseudomolecules sizes of about 40 Mbp (about 20 Mbp already present in Chr0 and 20 Mbp of completely new sequence) in this new assembly, which corresponds approximately to 11% of the total genome length. **Supplementary Table S2** reports the anchoring of 21,283 unassigned contigs of the v3.6.1 Chr0 (96.2%) on the new PacBio melon assembly. A synteny analysis of v3.6.1 and v4.0 assemblies showed a high degree of correspondence across all chromosomes, with short re-oriented or reordered blocks on all chromosomes except in Chr3, Chr8, and Chr11 (**Supplementary Figures S3** and **S4**). In terms of block relocation among chromosomes noteworthy changes were detected between Chr02, Chr11, and Chr12 of v4.0 assembly and Chr05, Chr06, Chr08, and Chr10 of the v3.6.1 assembly, respectively (**Supplementary Figure S3**).

In order to assess the level of completeness of the new assembly with respect to the gene content, we performed a BUSCO analysis. We obtained 94.8% of complete and 1.7% of fragmented BUSCOs at the genome level and a 91.1% of complete and 2.4% fragmented BUSCOs at the gene model level. The observed values are comparable to the ones reported in the v3.6.1 genome assembly, suggesting that the previous assembly has captured most of the gene information. To maintain gene models and names, the current annotation was transferred to the v4.0 PacBio assembly through a liftover process. We successfully moved 28,299 out of 29,980 gene models to the new genome assembly. The 5% (1,618) of transcripts that did not pass the MAKER's thresholds to define a proper gene model mainly consist of proteins with unknown function (651), transposons (79), and girdin-like proteins (39). In terms of distribution, about 22% of them (374) were from the unassembled contigs in Chr0. This failure could be due to the fact that part of these genes, especially those with unknown functions, may represent false or partial gene models in the previous genome annotation. The re-arrangements of some contigs in the new assembly may also be in part responsible of this discrepancy. A complete list of these genes is provided in **Supplementary Table S3**.

### Assembly v4.0 Captures a Larger Fraction of Repetitive Elements

We used the TEdenovo pipeline from the REPET package to identify TE sequences from the melon v4.0 assembly and to build TE consensuses. Subsequently, two iterations of TEannot were run to annotate TE sequences. Transposons cover 45.2% of the new genome assembly (excluding unclassified sequences), in comparison to 35.7% found in the v3.6.1 (Morata et al., 2018). Similarly to what was found in the v3.6.1 genome assembly, LTR-retrotransposons represented the largest fraction of TEs in the v4.0 assembly, followed by Terminal Inverted Repeats (TIRs) containing TEs (TIR-TEs) (**Table 1**). The number of LTR-retrotransposons is higher for v4.0 but the genome fraction that LTR-retrotransposons account for in the two assemblies is similar. On the contrary, we observed a drastic increase in the amount of annotated TIR-TEs and the genome fraction they account for in v4.0 as compared with v3.6.1 (14.97% and 7.11% respectively). We tested for TIR-TE copies that retained coding potential and found that in both cases the vast majority of the annotated elements were non-coding (89.1% in v3.6.1 and 96.1% in v4.0). The v4.0 assembly has 851 more coding TIR-TEs as compared with v3.6.1. However, the main difference between the TIR-TE fraction of both assemblies is explained by the differential amount of non-coding elements. The size distribution of TIR-TEs (**Supplementary Figure S5**) also supports this result, as the biggest differences between the two annotations are found for sizes between 100–500 bp, which are compatible with the length of MITEs and partial TE copies. These differences can be attributed in part to different annotation thresholds. Indeed, the peak found at 100 bp in v4.0 absent in v3.6.1 reflects a difference in the annotation approach (minimum annotation size = 200bp in v3.6.1). However, v4.0 contains more TIR-TEs elements of all sizes, and in particular of elements with a size shorter than 1,000 nt that probably represent truncated TIR-TEs and MITEs, which could be the result of a more complete representation of repetitive sequences in the assembly.

**Table 1:** Comparison of the transposable element (TE) annotation based on the v3.6.1 and v4.0 assemblies.

TE order	Acronym	v3.6.1		v4.0	
		Copies	Genome fraction (%)	Copies	Genome fraction (%)
LTR	RLX	74,161	23.44	136,761	23.81
LINE	RIX	11,913	2.64	15,067	1.80
SINE	RSX	391	0.04	746	0.04
DIRS	RYX	4,212	1.65	17,890	4.39
TIR	DTX	21,383	7.11	92,819	14.97
Helitron	DHX	1,699	0.3	5,637	0.45
Others		912	0.48	823	0.07
TOTAL			35.66		45.53

The TE orders are referred to as follows: LTR-retrotransposons (LTR), Long Interspersed nuclear elements (LINE), short interspersed nuclear elements (SINE), DIRS retrotransposons (DIRS), TIR-TEs (TIR), and Helitrons.

### Assembly v4.0 Contains Many Young LTR-Retrotransposons Missing In v3.6.1

**Figure 2:**
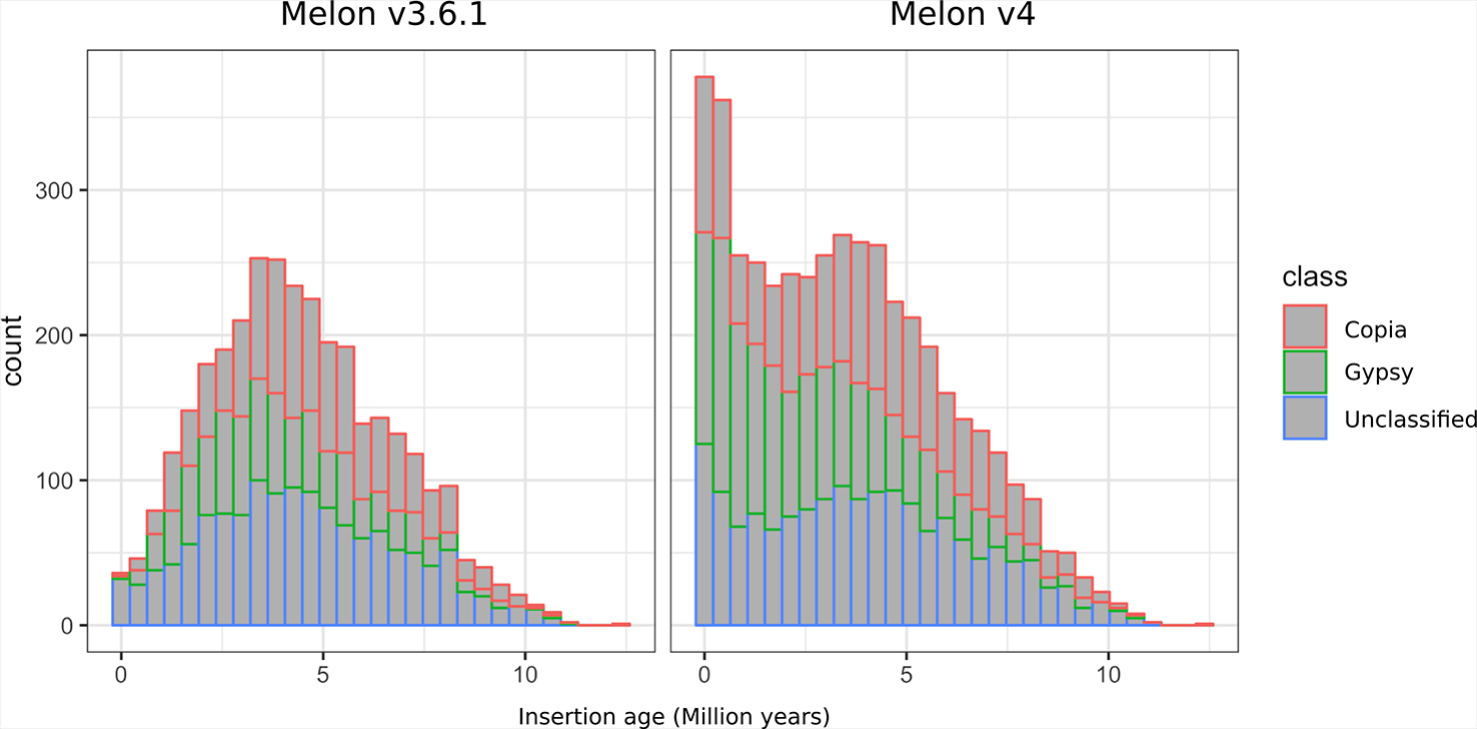
Distribution of insertion age of Gypsy, Copia, and unclassified LTR-retrotransposons annotated on the genome assemblies v3.6.1 and v4.0.

**Figure 3:**
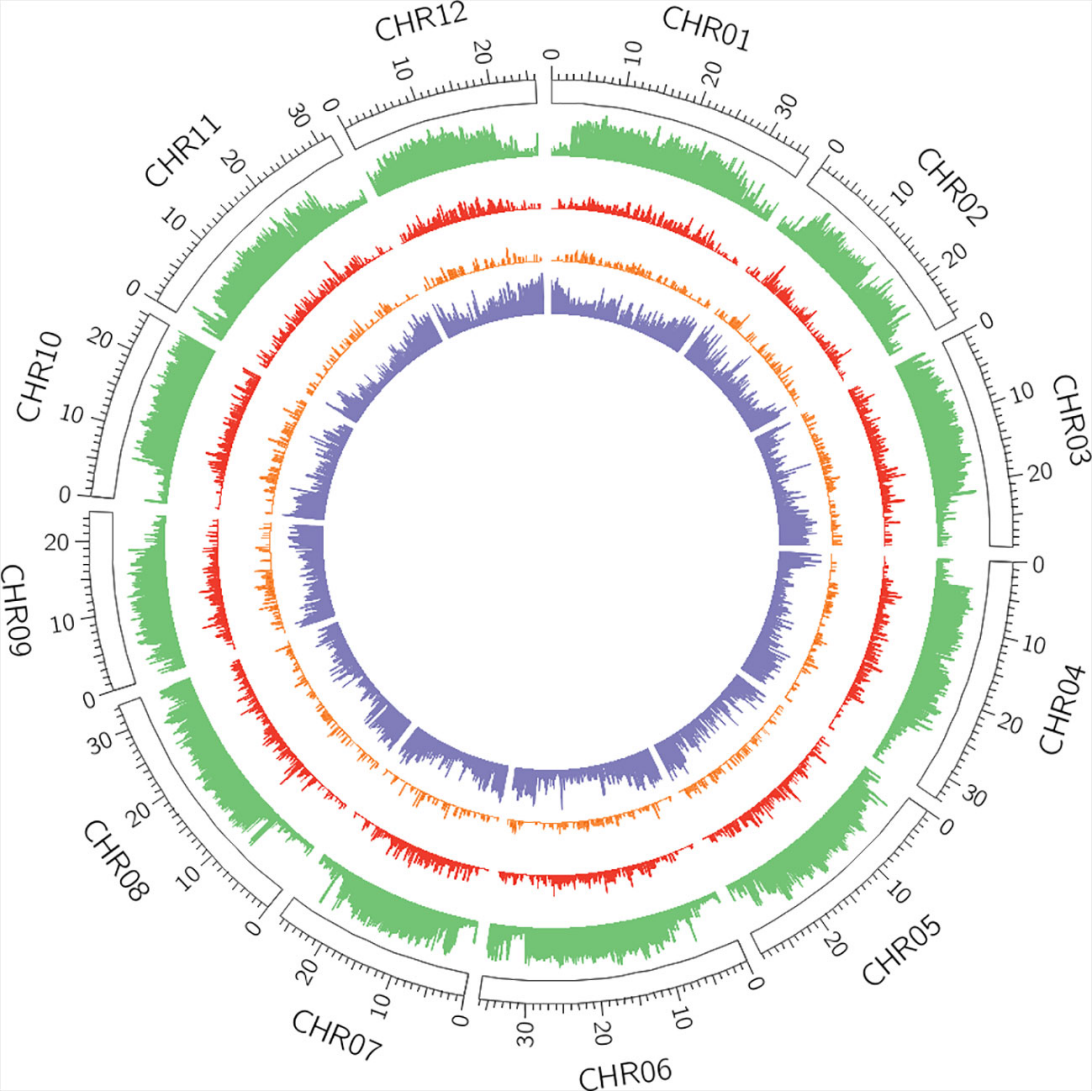
Distribution of transposable elements (TEs) and genes across v4.0 pseudomolecules. In green, density of REPET features per window (6,000 windows in total). In red, density of full-length Long Terminal Repeat (LTR)-retrotransposons annotated in the v4.0 that were absent in the v3.6.1 assembly. In orange, density of polymorphic LTR-retrotransposons with insertion time below 2 Mya. In purple, gene density.

Among the different classes of TEs that populate plant genomes, young LTR-retrotransposons are the most difficult to assemble due to their length and high similarity between copies. LTR-retrotransposons are frequently abundant and show a high level of polymorphism in varieties and individuals, which make them important targets of study. In order to annotate these elements in the v4.0 assembly and compare the LTR-retrotransposon content with that of v3.6.1, we used a structural and homology-based approach to identify and to date LTR-retrotransposon insertions. Using this approach, we annotated 1,320 full-length elements more in v4.0 than in v3.6.1, which represents an increase of 40% (**Table 2**). An important fraction of the new LTR-retrotransposons belongs to the Gypsy superfamily, but the v4.0 assembly also contains more Copia LTR-retrotransposons than the v3.6.1. We dated the insertion of all full-length LTR-retrotransposons, and the results showed that the vast majority of newly assembled elements in v4.0 are very young, with estimated insertion times from 0 to 2 Mya (**Figure 2**). The distribution of v4.0-specific LTR-retrotransposons followed the general distribution profile of all the annotated TEs, with an accumulation along the centromeres and pericentromeres in all the chromosomes (**Figure 3**), their abundance decreasing in gene-rich regions.

**Table 2:** Annotation of full-length LTR-retrotransposons. Number of full-length retrotransposon copies belonging to Gypsy, Copia, and unclassified superfamilies in the published v3.6.1 and the v4.0 genome assemblies.

Superfamily	v3.6.1	v4.0
Gypsy	815	1,526
Copia	1,067	1,427
Unclassified	1,358	1,607
TOTAL	3,240	4,560

### Young LTR-Retrotransposons Are Highly Polymorphic and Have a Potential Impact on Genes

**Figure 4:**
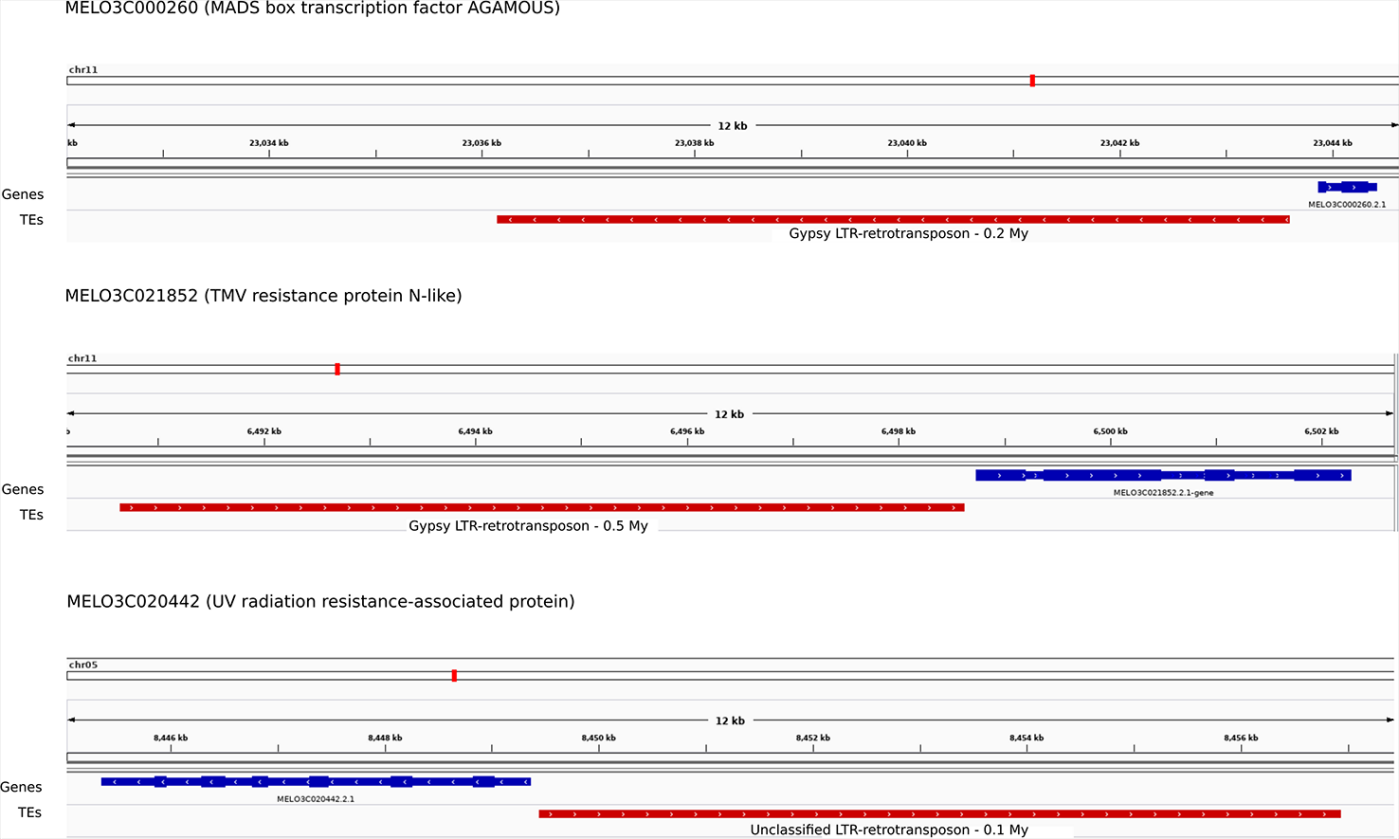
Example of LTR-retrotransposon insertion (red boxes) in the proximal upstream region of genes (blue boxes) annotated in the V4.0 assembly and that corresponded to a gap in the v3.6.1 assembly.

In order to determine to what extent the new assembled elements missing in v3.6.1 had a potential impact on genes, we analyzed in detail all the young, full-length LTR-retrotransposons (0-2 Mya). V3.6.1 contains 443 of these elements, whereas v4.0 contains up to 1,523. Using resequencing data from six varieties and the new v4.0 assembled genome as a reference, we were able to determine the level of polymorphism of these elements. More than half (777) of these young elements were predicted to be absent in at least one of the six varieties. The newly discovered young LRT-retrotransposons in the genome assembly v4.0 were further studied for their potential impact on genes. We found that 116 out of the 1,523 were located in the close upstream regions of annotated genes (< 1,000 bp, **Supplementary Table S4**), and therefore may be affecting the promoters of such genes. In addition, almost 60% of these elements (69) were predicted to be polymorphic in the six varieties analyzed (**Supplementary Table S5**). An example is the polymorphic Gypsy LTR-retrotransposon inserted into the promoter of an AGAMOUS MADS box transcription factor (MELO3C000260, **Figure 4**). This element is young (0.2 Mya), and predicted to be absent in 4 out of the 6 re-sequenced varieties. This Gypsy element could not be properly assembled in v3.6.1, which shows several gaps in the corresponding region upstream the AGAMOUS gene. In addition, in the v3.6.1 assembly the gene was located in the artificial Chr0, which contained the unassembled contigs, whereas in v4.0 assembly we could place it in Chr11 at the position 23,043,868 bp-23,044,427 bp. A manual inspection of this region allowed the correction of the AGAMOUS MADS box transcription factor gene by combining both MELO3C0002360 and MELO3C019694 (**Supplementary Figure S6**). Other examples of genes carrying a newly assembled LTR-retrotransposon absent in v3.6.1 are a TMV resistance protein N-like (MELO3C021852.2) and a UV radiation resistance-associated protein (MELO3C020442), among others (**Figure 4**, **Supplementary Table S4**). A gene ontology enrichment analysis found no enriched terms within the functional annotation of the 116 genes.

## Discussion

### An Improved Melon Reference Genome Assembly Produced Using Long-Read Sequencing

A high-quality and accurate reference genome represents a relevant resource for basic and applied research including functional genetics, comparative genomics, and population genetics (Kingan et al., 2019). Indeed, many reference genomes for crop plants have been generated over the past decade, even though most of them are often fragmented and missing complex repeat regions (Jiao et al., 2017b). Melon is a widely cultivated crop in the world, and its reference genome was first published in 2012 (Garcia-Mas et al., 2012). This reference genome has been improved over the time (Argyris et al., 2015; Ruggieri et al., 2018), and consists of 42,067 small contigs, assembled in 13 scaffolds. Some of the contigs were still arbitrarily ordered and oriented, which complicated the analysis of some individual loci. In addition, the last published version of the assembly, version v3.6.1, also contains a high number of short gaps, frequently found in intergenic regions and often close to genes. These drawbacks are a limitation for genotype to phenotype analyses, as gaps may contain sequence variability that cannot be used for GWAS or fine-mapping studies, and can also contain candidate genes that cannot be associated to the trait. On the other hand, finding significant SNPs scattered across unassigned scaffolds can complicate the interpretation of GWAS. All these limitations of incomplete assemblies for genotype to phenotype studies have been previously highlighted (International Wheat Genome Sequencing Consortium (IWGSC) et al., 2018; Benevenuto et al., 2019). Here, we combined PacBio (50x) with Illumina (20x) reads to improve the genome assembly v3.6.1 of the melon reference genome DHL92. The use of 2^nd^ generation Illumina sequencing technology to correct PacBio long reads is reported to be an efficient and cost effective way to improve a genome assembly (Mahmoud et al., 2019). This integrated workflow produced an improvement of the genome assembly both in terms of new sequence gained (20 Mbp) and inclusion of previous unassigned contigs (20 Mb). In addition, short blocks were reoriented or reordered within and across chromosomes. The structure of the genome is therefore improved in assembly version v4.0 presented here. The assessment of genome completeness and sequence accuracy of the v4.0 assembly was performed using a set of “Eudicotyledon” conserved genes. This analysis indicated that the main assembly improvements occurred in non-genic regions, in line with what has already been reported for other genomes (Jiao et al., 2017a). The number of gaps was reduced from 44,650 in the genome version v3.6.1 to 1,169 in v4.0 (**Supplementary Figure S7**). The remaining gaps probably result from the presence of very complex regions in the genome that will need further efforts to be solved.

### V4.0 Assembly Uncovers a Burst of Young LTR-Retrotransposons

The new v4.0 assembly of the melon genome contains up to 10% more TE content than v3.6.1, a difference that can be explained mainly by a better capture of coding and non-coding TIR-TEs, as well as a better identification of young LTR-retrotransposons. Non-coding TIR-TEs such as MITEs have been described to be involved in gene regulation through the amplification of transcription factor binding sites (TFBS) (Hénaff et al., 2014; Morata et al., 2018). Thus, our new dataset represents a significant improvement that can be used to assess the functional impact of these elements with a much better precision. Besides the importance of TIR-TEs, LTR-retrotransposons are the most interesting TEs due to their high abundance and their potential impact on genes. In this v4.0 assembly, the LTR-retrotransposon content is similar to that of v3.6.1 in percentage of genome fraction. However, we found a large difference in the content of full-length and young elements. Full LTR-retrotransposons are difficult to annotate with approaches that use genome self-comparison followed by RepeatMasker annotation (i.e., as REPET does). This approach can be effectively used to identify truncated and degenerated copies, but often leads to the fragmentation of long intact elements. To overcome this problem, a structural detection (LTRharvest) followed by a homology-based approach was used to identify full-length elements with coding potential in both v3.6.1 and v4.0 assemblies. Using the same annotation pipeline, we were able to identify up to 40% more full-length LTR-retrotransposons in the new v4.0 assembly, an important fraction of which are located in centromeric and pericentromeric regions. It is well known that Gypsy elements tend to integrate in such regions, which are highly repetitive and difficult to assemble. In this sense, this result evidences that v4.0 assembly captures a much larger fraction of the pericentromeres than v3.6.1 due to the improved assembly of LTR-retrotransposons, especially the younger ones. Our results evidence that a recent (less than 2 Mya) burst of LTR-retrotransposons occurred in the melon genome, which was overlooked in previous analysis due to the incompleteness of the reference assembly. Based on our results, we warn that comparisons of LTR-retrotransposon content and distribution between genome assemblies of very different quality could be strongly biased and should be carefully discussed.

### Impact of Young LTR-Retrotransposons on Genes

The genome of melon has been described to have recently expanded pericentromeric regions resulting from a massive TE amplification (Garcia-Mas et al., 2012). The number of young LTR-retrotransposons found in these regions supports the hypothesis that the pericentromeric expansion of melon occurred after the split with cucumber, which was dated about 10 Mya (Sebastian et al., 2010). Here, we have annotated a higher amount of young LTR-retrotransposons (< 2 Mya) located in pericentromeric regions providing an additional support to the hypothesis of the expansion of these regions through the accumulation of LTR-retrotransposon insertions. In addition to the important number of previously unassembled LTR-retrotransposons sitting in the pericentromeric regions, the v4.0 assembly also contains a high number of new LTR-retrotransposons in gene-rich regions. The detection of recent LTR-retrotransposon insertions at close proximity of genes (< 1 kb away) indicated potential to alter or regulate gene expression. In this study, 60% of these LTR-retrotransposons were first found to be polymorphic in the six melon varieties, providing probable association with phenotypic variation in melon species. Further studies should be addressed to demonstrate this hypothesis. One of these young Gypsy LTR-transposons is inserted into the promoter of the AGAMOUS MADS box transcription factor MELO3C019694, which was miss-annotated in assembly v3.6.1 (**Supplementary Figure S6**). Recently, MELO3C019694 has been suggested as the candidate gene for the presence of sutures trait after performing GWAS and bi-parental mapping experiments (Zhao et al., 2019), and the orthologous SHP1 and SHP2 in Arabidopsis regulates pod dehiscence in this plant (Liljegren et al., 2000). The insertion of a Gypsy element in the promoter of MELO3C019694 will have to be tested in a wide collection of non- and sutured accessions.

## Conclusion

We present here a new assembly of the melon genome, based on a combination of PacBio and Illumina sequencing, with an improved sequence content and continuity with respect to the previous published assembly version. The v4.0 genome assembly enables identification of important recent LTR-retrotransposon insertions at genes and their polymorphism among melon varieties. These insertions may affect the coding capacity or the expression of melon genes and may be linked to phenotypic variability in agronomic traits, as for example, the presence of sutures in the fruit.

## Data Availability Statement

The raw sequencing data and the assembly are available at the European Nucleotide Archive, ENA PRJEB34181. The assembly and the gene and TE annotations are available at the Melonomics database (www.melonomics.net).

## Author Contributions

JG-M and JC conceived the project. MP obtained the DNA. VR and RC obtained and analyzed the data. VR, RC, JG-M, and JC drafted the manuscript. All authors revised and approved the manuscript.

## Funding

This work was supported by the Spanish Ministry of Economy and Competitiveness grant AGL2015-64625-C2-1-R to JG-M and AGL2016-78992-R to JC, as well as Severo Ochoa Programme for Centres of Excellence in R&D 2016-2010 (SEV-2015-0533) and the CERCA Programme/Generalitat de Catalunya to both groups. RC was recipient of a Juan de la Cierva Postdoctoral fellowship from the Spanish Ministerio de Economia y Competitividad.

## Conflict of Interest

The authors declare that the research was conducted in the absence of any commercial or financial relationships that could be construed as a potential conflict of interest.
