# Supplementary material for: An Improved Melon Reference Genome With Single-Molecule Sequencing Uncovers a Recent Burst of Transposable Elements With Potential Impact on Genes
Source: Front Plant Sci. 2020 Jan 31;10:1815. doi: 10.3389/fpls.2019.01815 (PMC7006604; doi:10.3389/fpls.2019.01815)
Supplement: Supplementary file 1 [file Presentation_1.pptx]

## Slide 1
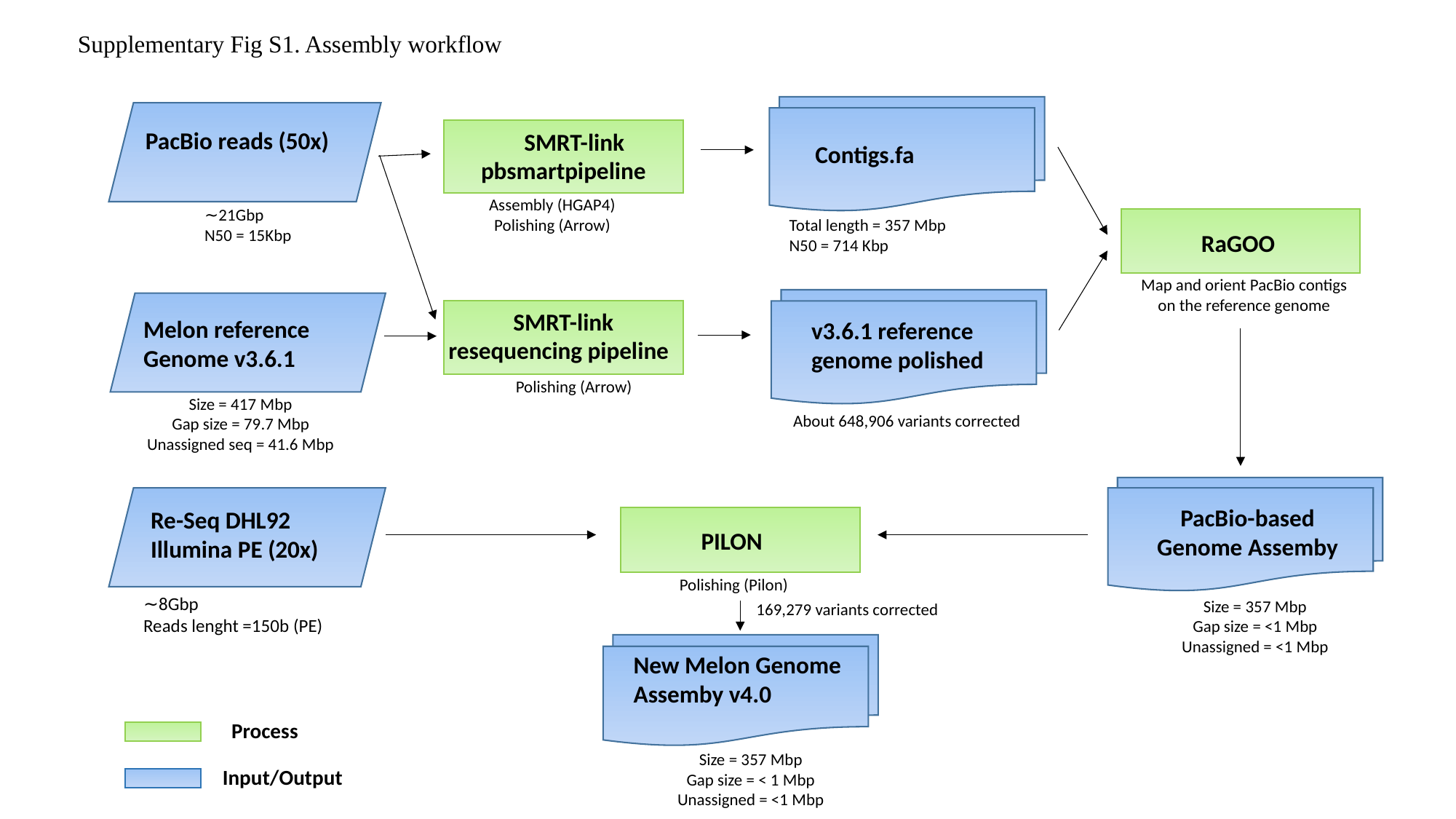

Supplementary Fig S1. Assembly workflow
 PacBio reads (50x)
SMRT-link
pbsmartpipeline
Contigs.fa
Assembly (HGAP4)
Polishing (Arrow)
∼21Gbp
N50 = 15Kbp
Total length = 357 Mbp
N50 = 714 Kbp
RaGOO
Map and orient PacBio contigs on the reference genome
SMRT-link
 resequencing pipeline
Melon reference Genome v3.6.1
v3.6.1 reference genome polished
Polishing (Arrow)
Size = 417 Mbp
Gap size = 79.7 Mbp
Unassigned seq = 41.6 Mbp
About 648,906 variants corrected
PacBio-based Genome Assemby
Re-Seq DHL92
Illumina PE (20x)
PILON
Polishing (Pilon)
∼8Gbp
Reads lenght =150b (PE)
Size = 357 Mbp
Gap size = <1 Mbp
Unassigned = <1 Mbp
169,279 variants corrected
New Melon Genome Assemby v4.0
Process
Size = 357 Mbp
Gap size = < 1 Mbp
Unassigned = <1 Mbp
Input/Output
