# Supplementary material for: An Improved Melon Reference Genome With Single-Molecule Sequencing Uncovers a Recent Burst of Transposable Elements With Potential Impact on Genes
Source: Front Plant Sci. 2020 Jan 31;10:1815. doi: 10.3389/fpls.2019.01815 (PMC7006604; doi:10.3389/fpls.2019.01815)

Supplementary Fig 2. A) Distribution of the coverage across the final assembly, B) distribution of the coverage across the contig 0004F, C) realigned subread concordance and D) realigned concordance vs. subread length.

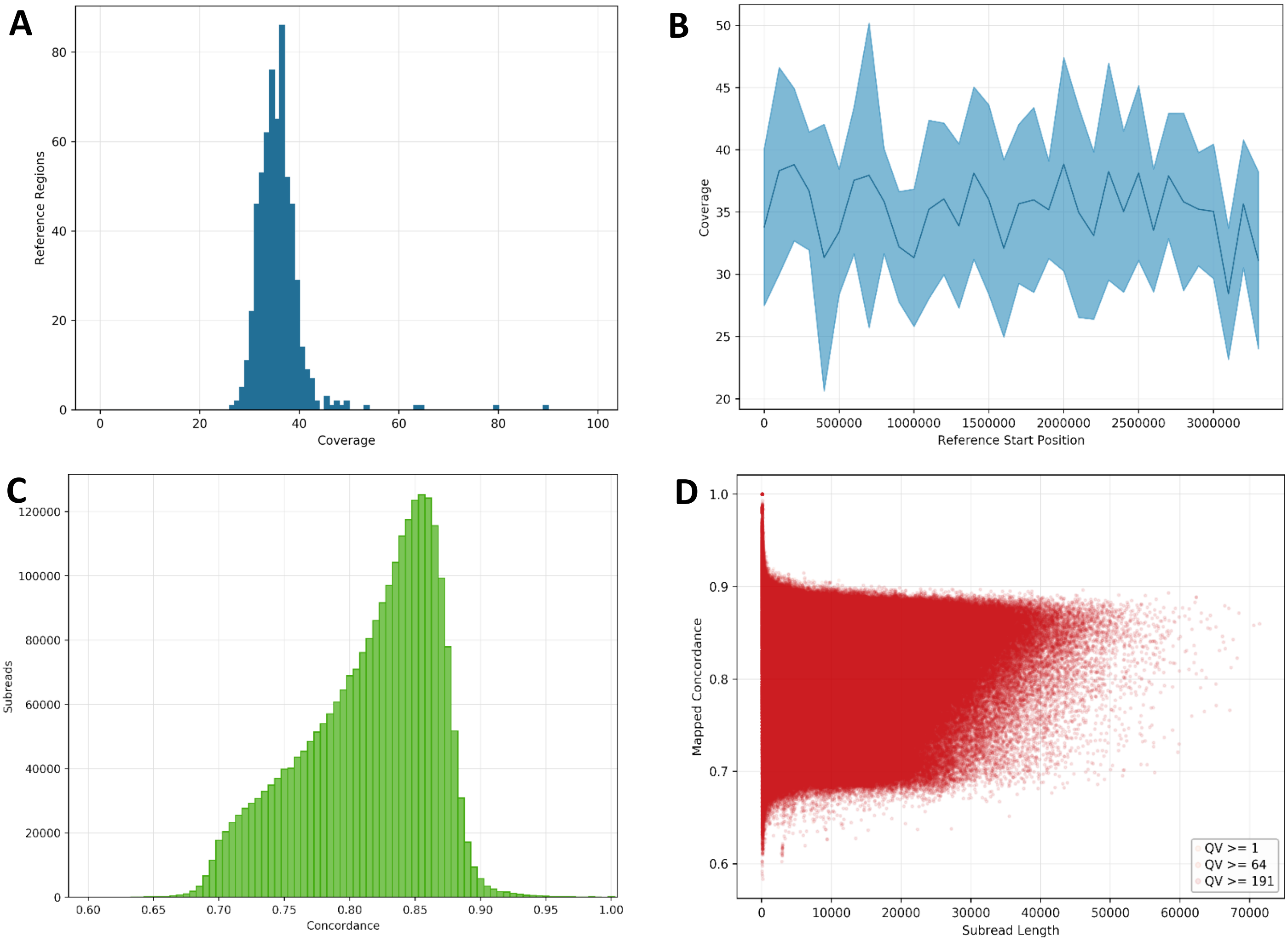

Supplement: Supplementary file 2 [file Presentation_2.pdf]
