# Supplementary material for: An Improved Melon Reference Genome With Single-Molecule Sequencing Uncovers a Recent Burst of Transposable Elements With Potential Impact on Genes
Source: Front Plant Sci. 2020 Jan 31;10:1815. doi: 10.3389/fpls.2019.01815 (PMC7006604; doi:10.3389/fpls.2019.01815)
Supplement: Supplementary file 3 [file Presentation_3.pptx]

## Slide 1
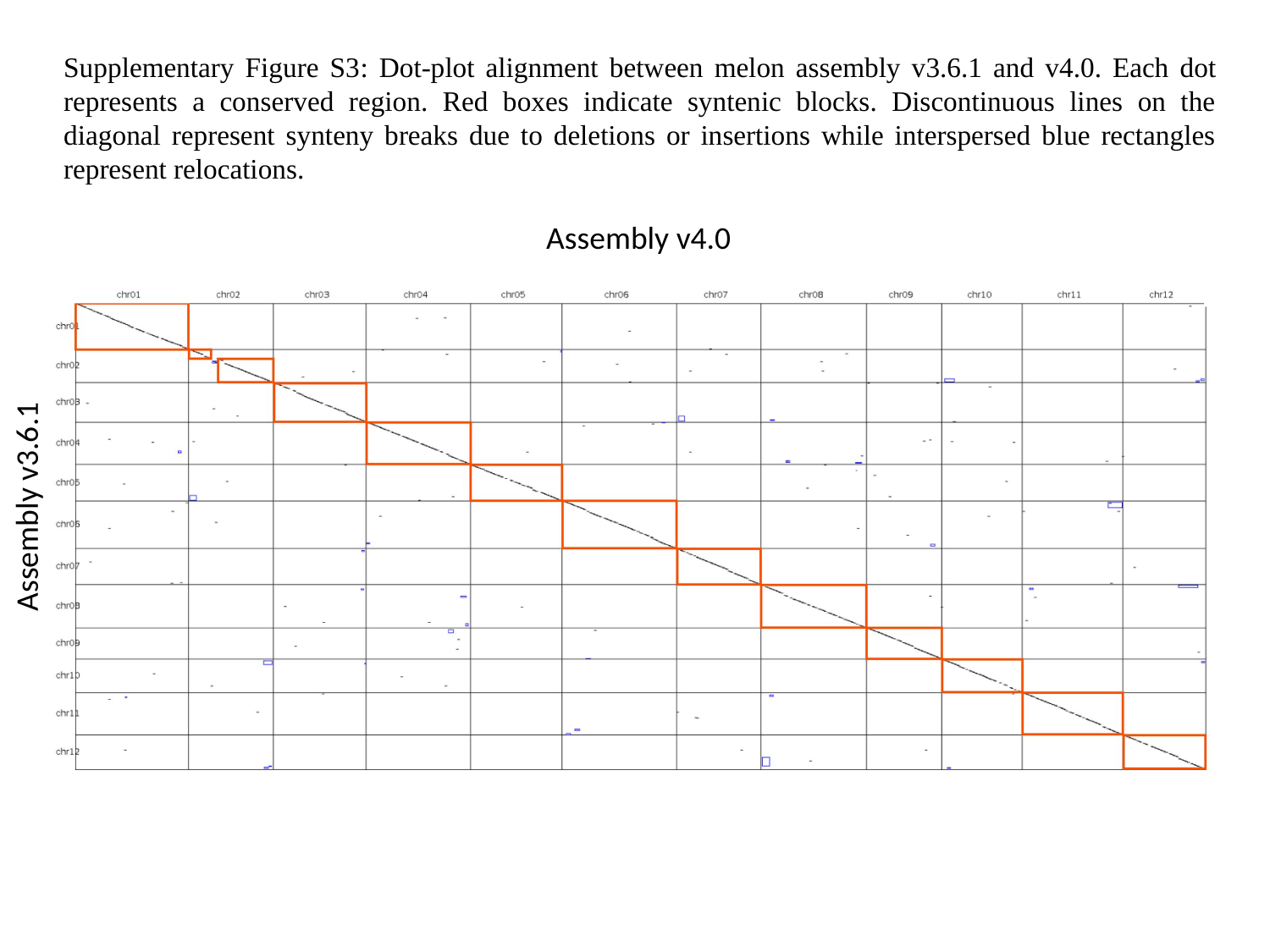

Supplementary Figure S3: Dot-plot alignment between melon assembly v3.6.1 and v4.0. Each dot represents a conserved region. Red boxes indicate syntenic blocks. Discontinuous lines on the diagonal represent synteny breaks due to deletions or insertions while interspersed blue rectangles represent relocations.
Assembly v4.0
Assembly v3.6.1
