# Supplementary material for: An Improved Melon Reference Genome With Single-Molecule Sequencing Uncovers a Recent Burst of Transposable Elements With Potential Impact on Genes
Source: Front Plant Sci. 2020 Jan 31;10:1815. doi: 10.3389/fpls.2019.01815 (PMC7006604; doi:10.3389/fpls.2019.01815)
Supplement: Supplementary file 4 [file Presentation_4.pptx]

## Slide 1
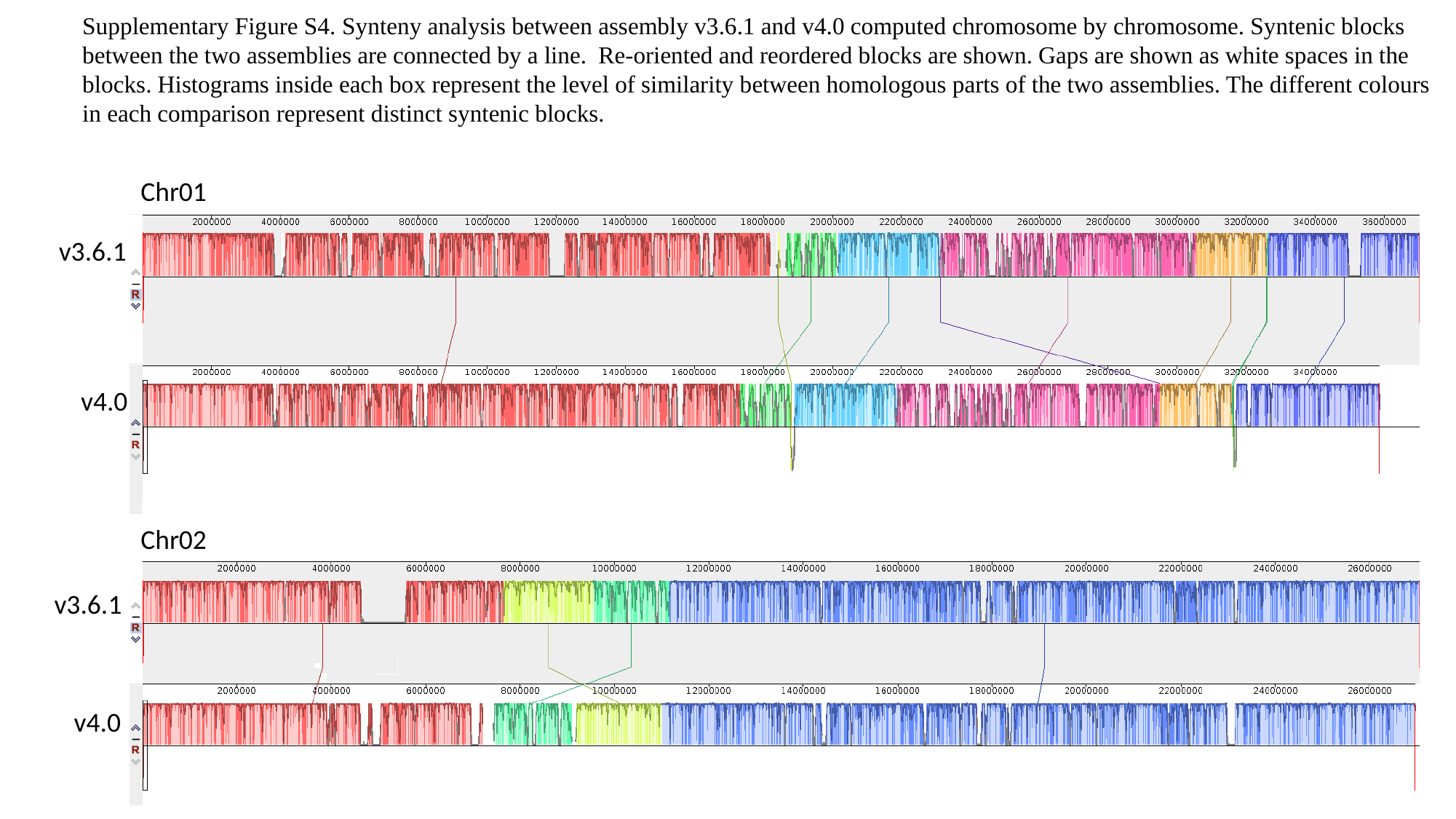

Supplementary Figure S4. Synteny analysis between assembly v3.6.1 and v4.0 computed chromosome by chromosome. Syntenic blocks between the two assemblies are connected by a line. Re-oriented and reordered blocks are shown. Gaps are shown as white spaces in the blocks. Histograms inside each box represent the level of similarity between homologous parts of the two assemblies. The different colours in each comparison represent distinct syntenic blocks.
Chr01
v3.6.1
v4.0
Chr02
v3.6.1
v4.0

## Slide 2
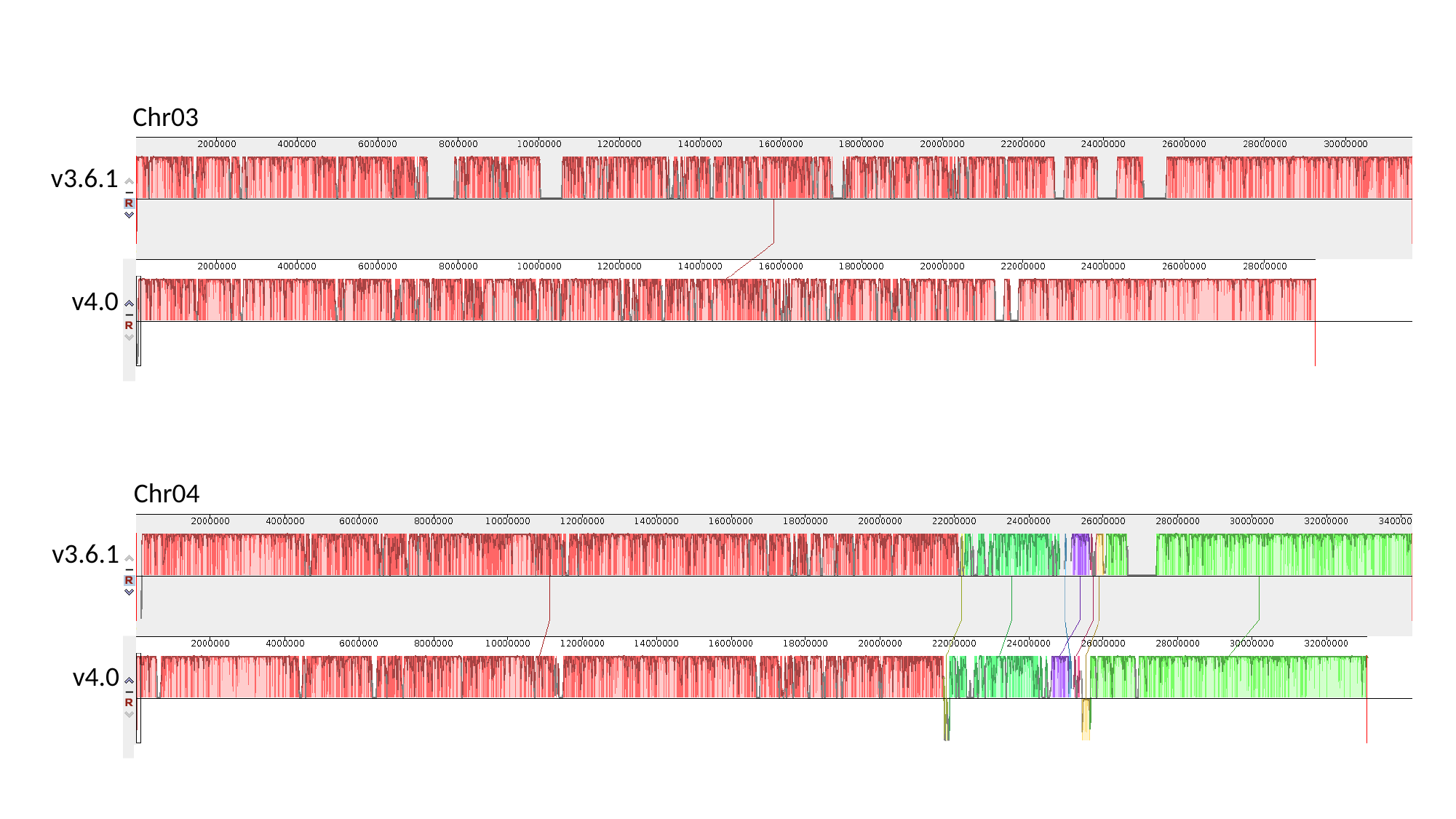

Chr03
v3.6.1
v4.0
Chr04
v3.6.1
v4.0

## Slide 3
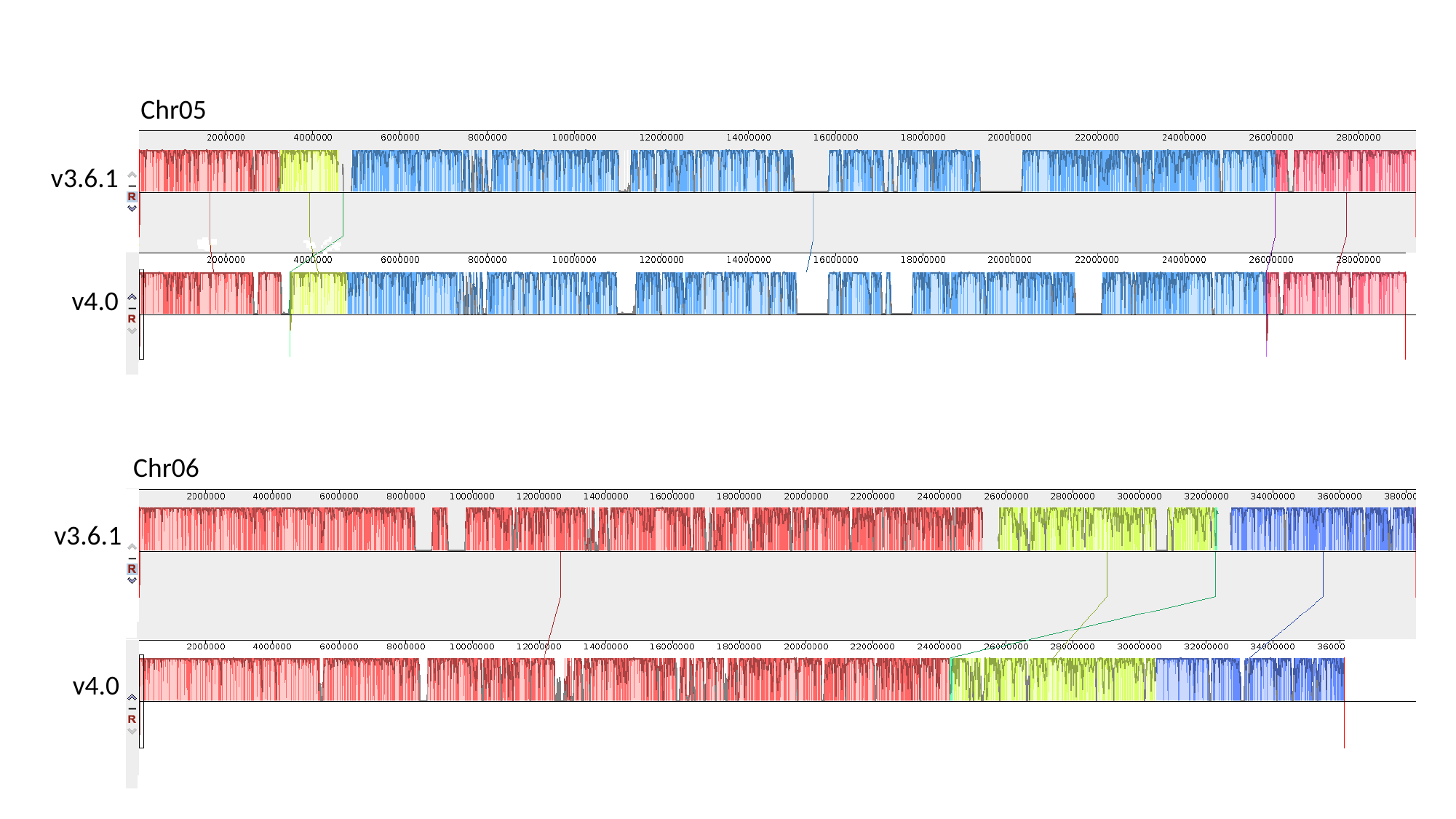

Chr05
v3.6.1
v4.0
Chr06
v3.6.1
v4.0

## Slide 4
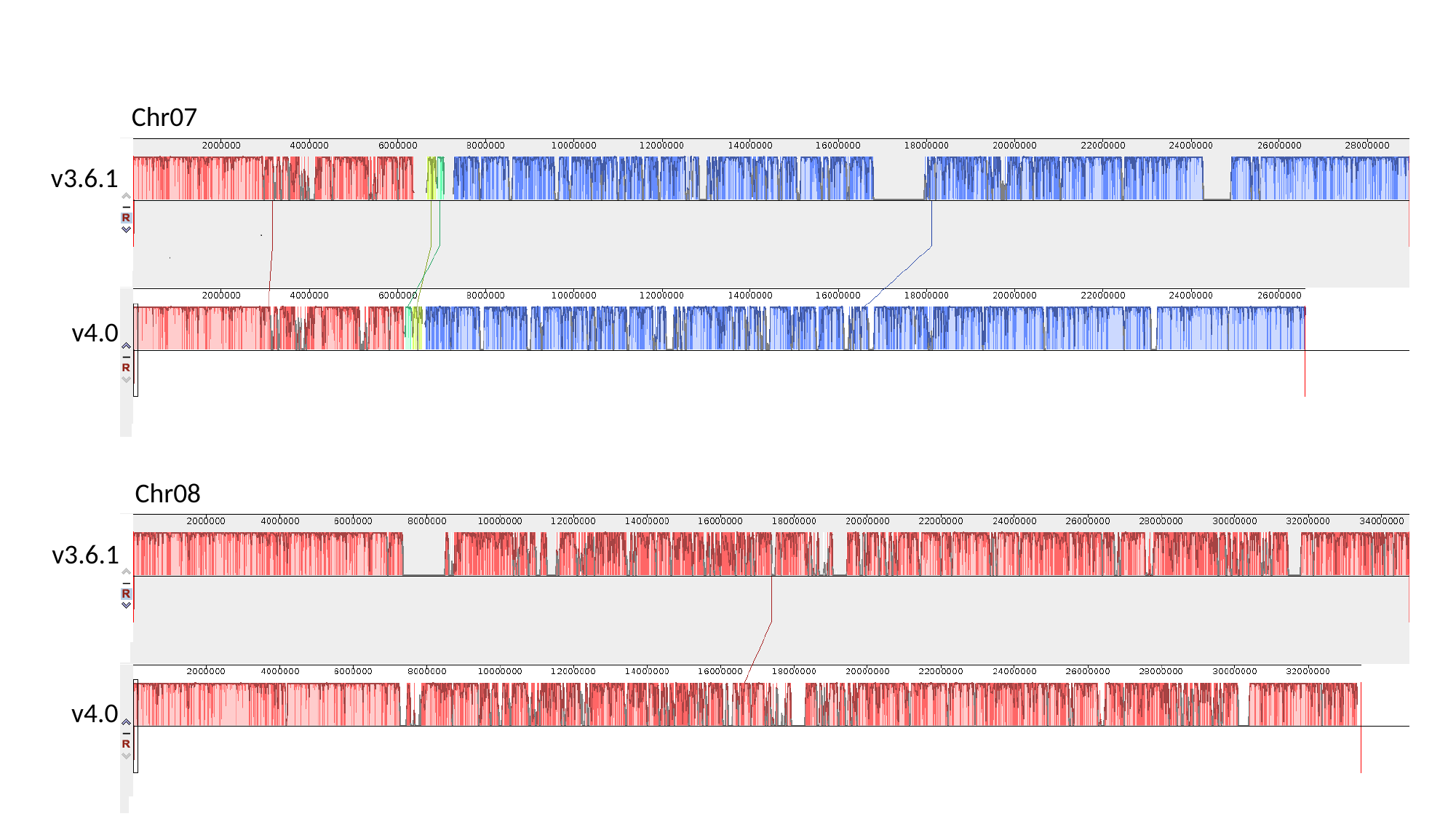

Chr07
v3.6.1
v4.0
Chr08
v3.6.1
v4.0

## Slide 5
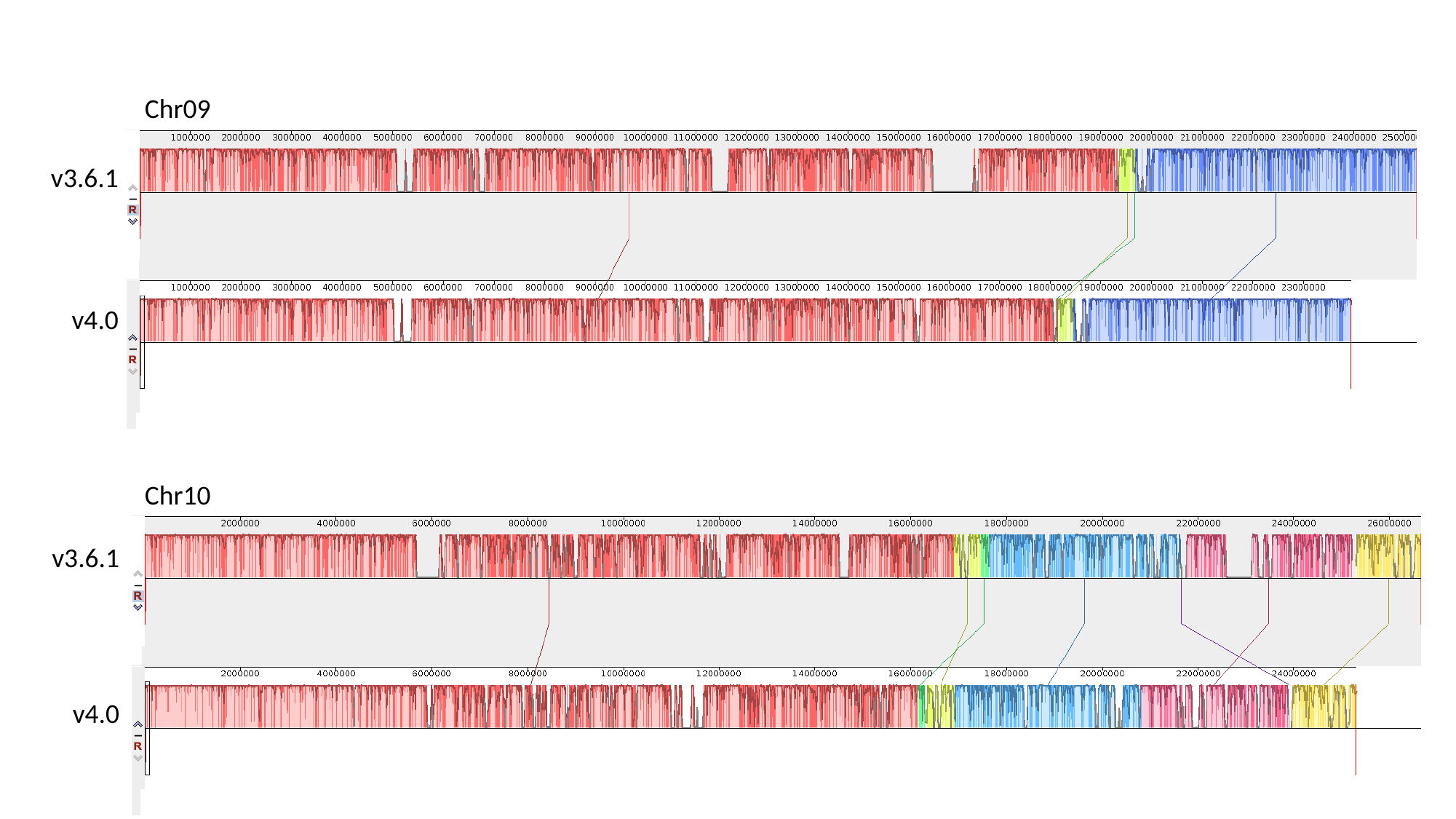

Chr09
v3.6.1
v4.0
Chr10
v3.6.1
v4.0

## Slide 6
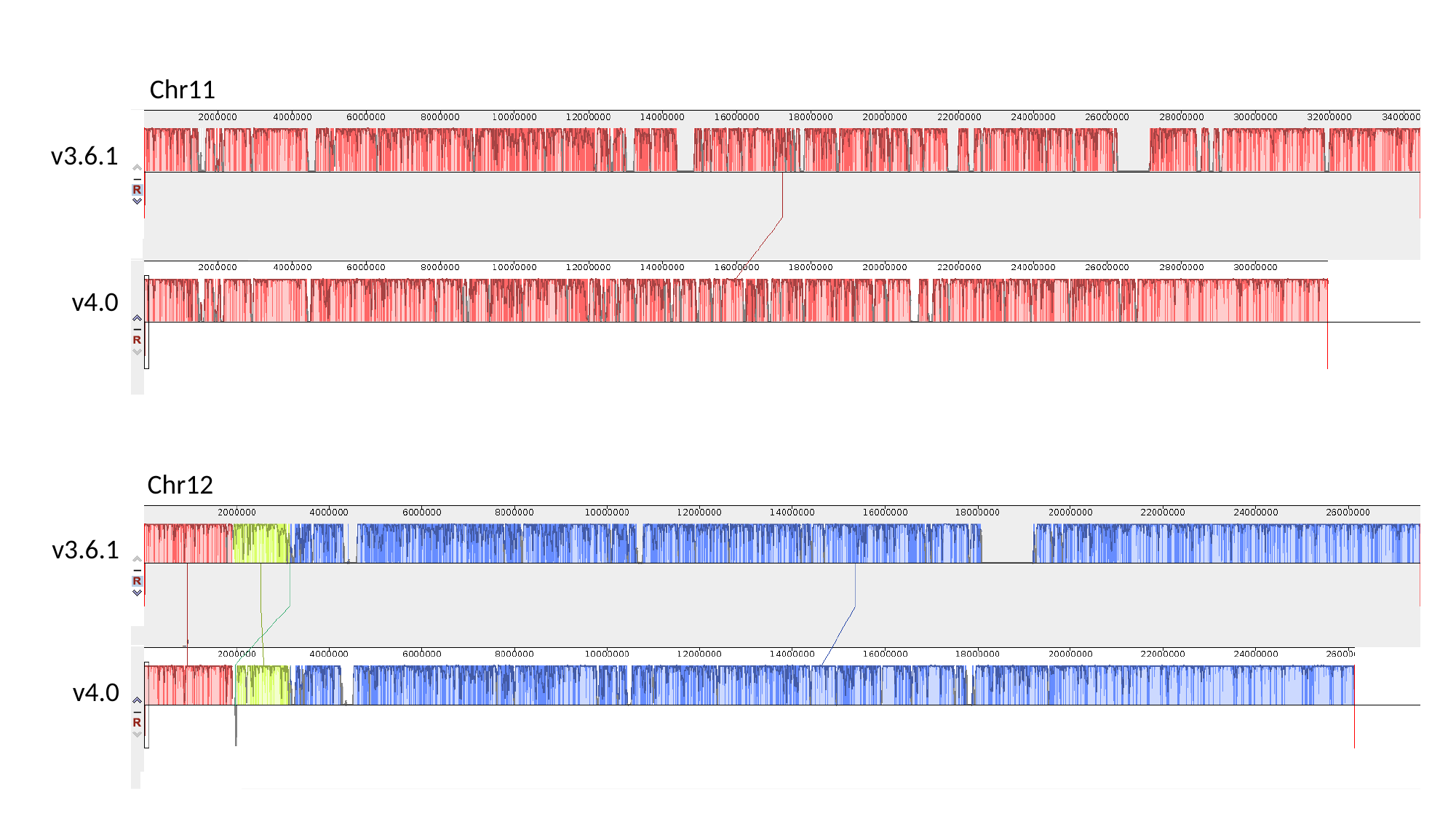

Chr11
v3.6.1
v4.0
Chr12
v3.6.1
v4.0
