# Supplementary figures and images for: An Improved Melon Reference Genome With Single-Molecule Sequencing Uncovers a Recent Burst of Transposable Elements With Potential Impact on Genes
Source: Front Plant Sci. 2020 Jan 31;10:1815. doi: 10.3389/fpls.2019.01815 (PMC7006604; doi:10.3389/fpls.2019.01815)

## Slide 1
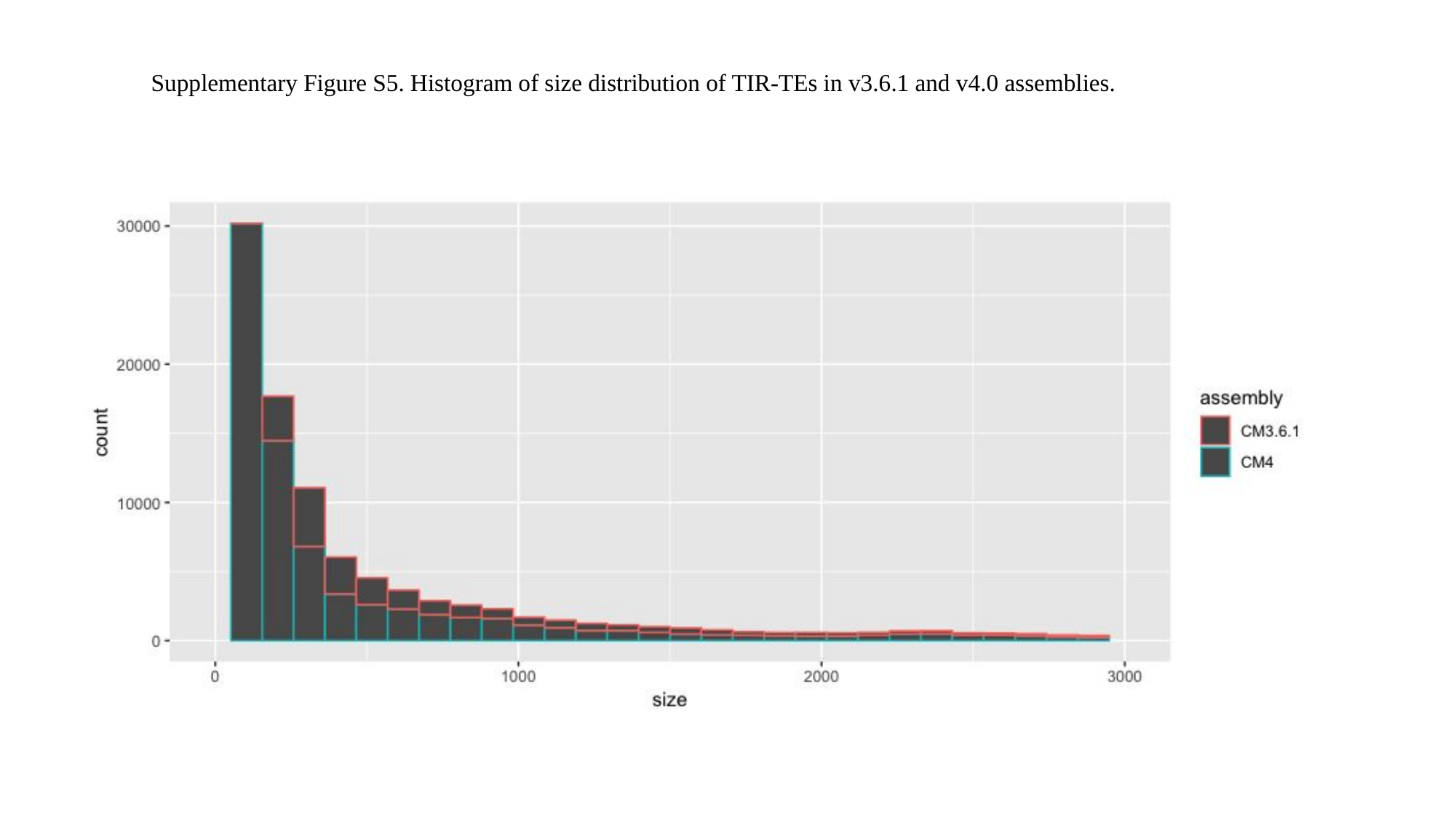

Supplementary Figure S5. Histogram of size distribution of TIR-TEs in v3.6.1 and v4.0 assemblies.

Supplement: Supplementary file 5 [file Presentation_5.pptx]
