# Supplementary material for: An Improved Melon Reference Genome With Single-Molecule Sequencing Uncovers a Recent Burst of Transposable Elements With Potential Impact on Genes
Source: Front Plant Sci. 2020 Jan 31;10:1815. doi: 10.3389/fpls.2019.01815 (PMC7006604; doi:10.3389/fpls.2019.01815)
Supplement: Supplementary file 6 [file Presentation_6.pptx]

## Slide 1
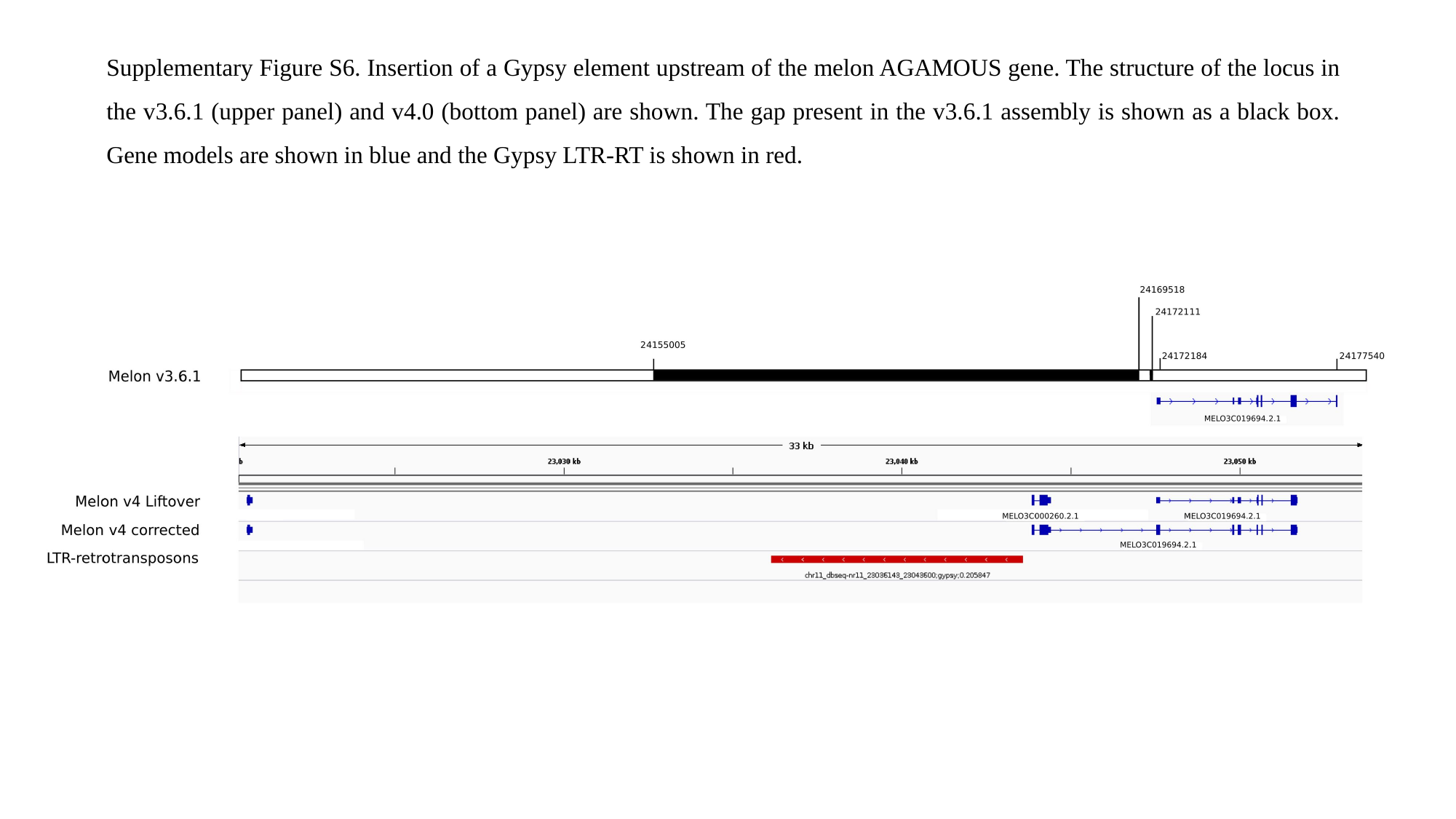

Supplementary Figure S6. Insertion of a Gypsy element upstream of the melon AGAMOUS gene. The structure of the locus in the v3.6.1 (upper panel) and v4.0 (bottom panel) are shown. The gap present in the v3.6.1 assembly is shown as a black box. Gene models are shown in blue and the Gypsy LTR-RT is shown in red.
