# Supplementary material for: An Improved Melon Reference Genome With Single-Molecule Sequencing Uncovers a Recent Burst of Transposable Elements With Potential Impact on Genes
Source: Front Plant Sci. 2020 Jan 31;10:1815. doi: 10.3389/fpls.2019.01815 (PMC7006604; doi:10.3389/fpls.2019.01815)

## Slide 1
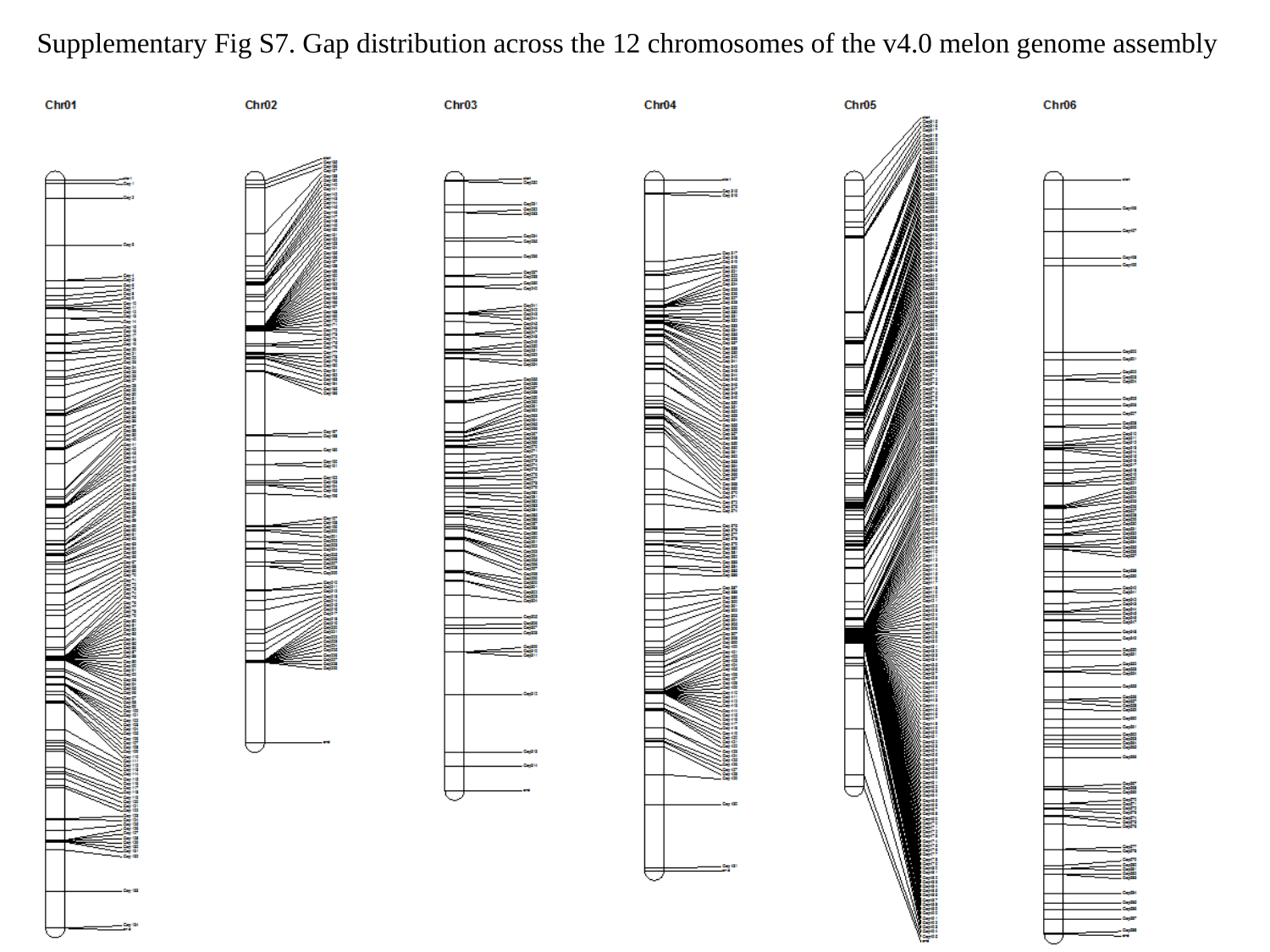

Supplementary Fig S7. Gap distribution across the 12 chromosomes of the v4.0 melon genome assembly

## Slide 2
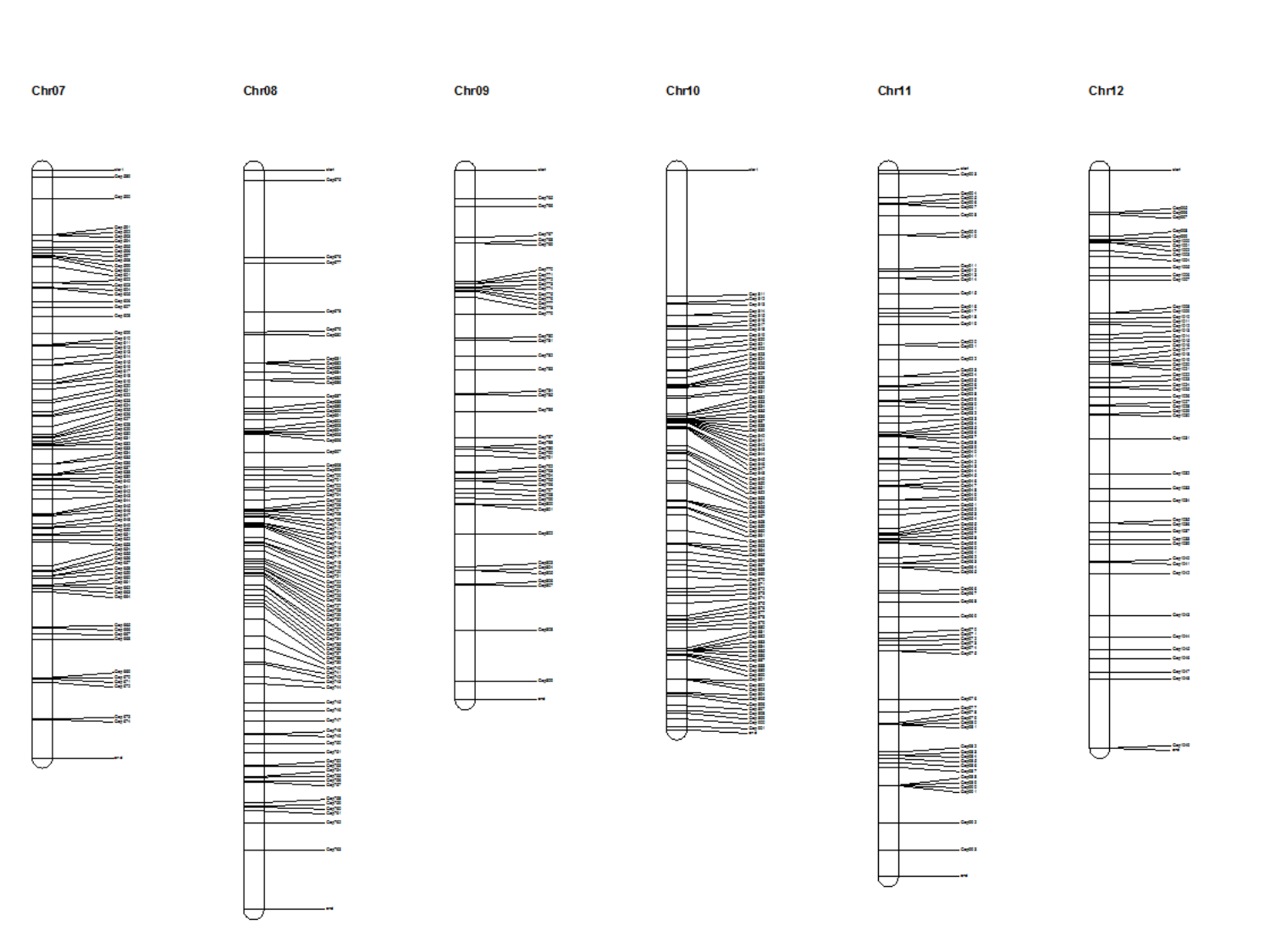

Supplement: Supplementary file 7 [file Presentation_7.pptx]
