# Supplementary material for: An Improved Melon Reference Genome With Single-Molecule Sequencing Uncovers a Recent Burst of Transposable Elements With Potential Impact on Genes
Source: Front Plant Sci. 2020 Jan 31;10:1815. doi: 10.3389/fpls.2019.01815 (PMC7006604; doi:10.3389/fpls.2019.01815)
Supplement: Supplementary file 9 [file Table_2.docx]

Supplementary Table S2. Anchoring of 21,283 unassigned v3.6.1 contigs (Chr0) to the chromosomes of v4.0 assembly.

|  | **Number of contigs with:** | | |  |
| --- | --- | --- | --- | --- |
| **chromosome (v4.0)** | **Identity > 99%** | **90 < Identity < 99** | **Identity < 90%** | **Total** |
| **chr00** | 7 | 16 | 3 | 26 |
| **chr01** | 1596 | 714 | 49 | 2359 |
| **chr02** | 920 | 416 | 61 | 1397 |
| **chr03** | 1120 | 458 | 20 | 1598 |
| **chr04** | 1068 | 546 | 18 | 1632 |
| **chr05** | 1461 | 755 | 18 | 2234 |
| **chr06** | 1447 | 567 | 17 | 2031 |
| **chr07** | 942 | 486 | 24 | 1452 |
| **chr08** | 1581 | 620 | 34 | 2235 |
| **chr09** | 861 | 399 | 40 | 1300 |
| **chr10** | 974 | 554 | 18 | 1546 |
| **chr11** | 1240 | 620 | 24 | 1884 |
| **chr12** | 994 | 511 | 84 | 1589 |
| **unassigned** |  |  |  | 840 |
| **Total** | 14211 | 6662 | 410 | 22123 |
